# Effect of Tea Catechins on Influenza Infection and the Common Cold with a Focus on Epidemiological/Clinical Studies

**DOI:** 10.3390/molecules23071795

**Published:** 2018-07-20

**Authors:** Daisuke Furushima, Kazuki Ide, Hiroshi Yamada

**Affiliations:** 1 Department of Drug Evaluation & Informatics, School of Pharmaceutical Sciences, University of Shizuoka, Shizuoka 422-8002, Japan; ide.kazuki.2r@kyoto-u.ac.jp; 2 Department of Pharmacoepidemiology, Graduate School of Medicine and Public Health, Kyoto University, Kyoto 606-8501, Japan; 3 Center for the Promotion of Interdisciplinary Education and Research, Kyoto University, Kyoto 606-8501, Japan

**Keywords:** tea catechins, influenza infection, common cold, bioactivity, epidemiological study

## Abstract

Influenza and the common cold are acute infectious diseases of the respiratory tract. Influenza is a severe disease that is highly infectious and can progress to life-threating diseases such as pneumonia or encephalitis when aggravated. Due to the fact that influenza infections and common colds spread easily via droplets and contact, public prevention measures, such as hand washing and facial masks, are recommended for influenza prophylaxis. Experimental studies have reported that tea catechins inhibited influenza viral adsorption and suppressed replication and neuraminidase activity. They were also effective against some cold viruses. In addition, tea catechins enhance immunity against viral infection. Although the antiviral activity of tea catechins has been demonstrated, the clinical evidence to support their utility remains inconclusive. Since the late 1990s, several epidemiological studies have suggested that the regular consumption of green tea decreases influenza infection rates and some cold symptoms, and that gargling with tea catechin may protect against the development of influenza infection. This review briefly summarizes the effect of tea catechins on influenza infection and the common cold with a focus on epidemiological/clinical studies, and clarifies the need for further studies to confirm their clinical efficacy.

## 1. Introduction

Influenza and the common cold are acute infectious diseases of the respiratory tract. Influenza, in particular, is a severe disease and presents a high morbidity and mortality burden on the population because it is highly infectious and can progress to life-threating diseases such as pneumonia or encephalitis when aggravated. The World Health Organization (WHO) reported annual infection rates of 5–10% among adults and 20–30% among children, and WHO also reported that more than 250,000 individuals die each year from a severe influenza-related illness such as pneumonia or encephalitis [1,2,3]. At least 14 pandemics occurred within the last 500 years before the global H1N1 pandemic in 2009 (H1N1pdm09) [4]. Influenza and the common cold spread easily via droplets and contact, and public prevention measures, such as hand washing and facial masks, are recommended for influenza prophylaxis. However, the beneficial effects of these non-pharmaceutical preventive measures are limited and not completely effective [5,6]. Therefore, new approaches for the prevention and treatment of influenza infections and the common cold are needed [7]. Recently, the preventative effect of catechin compounds contained in green tea against influenza infection and the common cold is attracting attention [8]. Catechins are a class of polyphenolic flavonoids that are included in tea leaves [9,10]. Tea leaves include (−)epigallocatechin gallate (EGCG), (−)epigallocatechin (EGC), (−)epicatechin gallate (ECG), (−)epicatechin (EC), and (+)catechin (C) [11]. Lin et al. [9] reported the following mean concentrations of total catechins among 13 types of Japanese and 15 types of Chinese green tea products: 17.80 mg/100 mg (13.74 for EGCG, 0.88 for EGC, 1.95 for ECG, 0.87 for EC) and 17.86 mg/100 mg (13.37 for EGCG, 0.44 for EGC, 2.91 for ECG, 0.55 for EC), respectively. The present review briefly summarizes the probable clinical inhibitory effects of tea catechins against influenza infection and common cold based on the results reported in clinical/epidemiological studies and highlights the benefits of catechin compounds.

## 2. Infection and Replication of the Influenza Virus

Influenza viruses are classified as type A, B, or C, of which types A and type B cause symptoms such as fever, muscle pain, chills, and cough [1]. They are also epidemiologically the most important for humans since they can recombine their genes with those of strains circulating animals, such as birds, swine, and horses. Conversely, the type C virus causes less severe symptoms and does not generally lead to influenza epidemics or pandemics. Type A and B viruses infect mucous membrane cells in the nose through two proteins, hemagglutinin (HA) and neuraminidase (NA), which are present as spikes on the surface of the virus particle [12]. According to a report by the Centers for Disease Control and Prevention (CDC) in the United States, 18 HA (from H1 to H18) and 11 NA (from N1 to N11) proteins are known, and the severe Spanish flu (1918) and Hong Kong flu (1968) pandemics were caused by the genesis of H1N1 and H3N2 subtypes, respectively. On the other hand, the type B virus is not classified into subtypes like the type A virus; instead, it is classified as a lineage and strain. Currently, two distinct type B virus lineages are circulating; B/Yamagata and B/Victoria [13]. The mechanism from influenza infection to onset is that, after the attachment of the influenza virus to host cells, the HA protein of the virus is recognized by *N*-acetylneuraminic acid in the host cell membrane. After the binding process, the virus enters the host cell and targets the perinuclear region via endosomal trafficking. Here, the endosomal membrane fuses with the virus resulting in its uncoating. The uncoated viral genome is then transported to the nucleus, where virus replication, assembly, and budding occur, and NA mediates the enzymatic cleavage of the viral receptor to release the progeny viruses [14,15].

## 3. Activity of Tea Catechins against the Influenza Virus

Catechins have been reported as potential anti-influenza virus agents in several experimental studies. An in vitro study showed that EGCG, the most abundant catechin in green tea, was shown to minimize the infectivity of the influenza A and B virus in Madin–Darby canine kidney cells [16,17]. Furthermore, EGCG and ECG inhibited the activity of viral RNA (ribonucleic acid), which suppressed virus propagation [18,19].

Catechins do not interfere with HA and NA functions; rather, they inhibit the interaction of a virus with the cell membrane when it invades a cell [20]. An animal experiment using chickens showed that a diet and water containing green tea components suppressed the replication of the influenza virus [21]. Furthermore, EGCG, ECG, and catechin-5-gallate have neuraminidase inhibitory activity, as demonstrated by a molecular docking study [22]. Also, the results of a molecular structural study showed that the interaction energy order, which is consistent with the effect of HA inhibition, was EGCG, ECG, EGC, and EC [23]. It has also been reported that EGCG exhibits broad-spectrum antiviral efficacy against various viral families, such as Flaviviridae, Retroviridae, Hepadnaviridae, Herpesviridae, Adenoviridae, Orthomyxoviridae, and Picornaviridae [16].

Several studies have reported that polyphenols enhance immune function [24,25,26]. An increase in natural killer (NK) cell activity and cytokine levels was observed in mice fed polyphenol-rich cereals [27]. Additionally, the administration of water-containing tea catechins maintained NK cell activity and prevented tumor metastasis in senescence-accelerated mice under an experimental setting [24]. Other studies regarding effectiveness of catechins against antivirals in fundamental studies are described in detail elsewhere [28,29]. Investigations of the antiviral effects of chemically-modified catechin derivatives have also been progressing in recent years [30,31].

## 4. Epidemiological/Clinical Studies on the Ability of Tea Catechins to Prevent Influenza Infection

The ability of catechins or natural tea compounds to prevent an influenza infection has been documented since the late 1990s [32,33,34]. Although experimental studies showed antiviral activity with tea consumption, evidence for its clinical efficacy is not conclusive. One epidemiological study focused on the association between green tea consumption habits and influenza infection, two studies assessed the effect of green tea based on dietary supplement consumption, and another five studies evaluated the effect of gargling with green tea on the prevention of influenza infection. The characteristics and findings of these studies are summarized in Table 1.

Park et al. [35] examined the effect of green tea consumption habits on preventing influenza infection by a cross-sectional study using a questionnaire. Their study was conducted in several primary schools in Shizuoka prefecture, Japan, and included 2050 children who returned the questionnaire. They revealed that there was an inverse association between 1–5 cups per day (1 cup = 200 mL) of green tea consumption and influenza infection (1–3 cups per day vs. <1 cup per day (reference), the adjusted odds ratio (OR) was 0.62 and the 95% confidence interval (95% CI) was 0.41–0.95; 3–5 cups per d vs. <1 cup per d, adjusted OR 0.54; 95% CI 0.30–0.94). In comparison, Rowe et al. [36] conducted a randomized controlled study using green tea capsules containing L-theanine and EGCG that equaled 10 cups of green tea per day and compared its consumption to that of a placebo. They enrolled 108 healthy adult participants that were randomly allocated to the green tea capsule group (N = 53) or the placebo group (N = 55), and they were followed for five months. The major outcome measure was presence of a cold or influenza symptoms. There were 32.1% fewer symptoms (*p* = 0.035) and 35.6% fewer symptom days (*p* < 0.002) in the green tea capsule group compared to the placebo group. Similarly, Matsumoto et al. [37] conducted a randomized controlled study using green tea capsules containing 378 mg per day of catechins that included 270 mg per day of EGCG and 210 mg per day of theanine and compared its consumption to that of a placebo. They enrolled 197 participants that were randomly allocated to the green tea capsule group (N = 98) or the placebo group (N = 99), and they were followed for three months. They found that the incidence of clinically diagnosed influenza infection was significantly lower in the catechin group (4.1%) compared with the placebo group (13.1%) (adjusted OR 0.25; 95% CI 0.07–0.76). In addition, the time for which the patient was free from influenza infection was significantly different between the groups (adjusted hazard ratio 0.27; 95% CI 0.09–0.84).

## 5. Prevention of Influenza Infection and Common Cold by Gargling with Tea Catechins

There are several reports on prevention of influenza infection or common cold by gargling with tea catechins; however, both are epidemiological studies conducted in Japan. Gargling, which is washing one’s mouth and throat with a liquid kept in motion by breathing through it with a gurgling sound, does not seem to be a common practice in European and North American countries. However, in Eastern Asian countries, especially in Japan, gargling is a generally accepted traditional intervention used for preventing upper respiratory tract infections such as influenza and the common cold [8,38].

Two observational studies and three randomized controlled studies were conducted to determine the efficacy of gargling with green tea (Table 2). The observational study by Noda et al. [39] assessed the effectiveness of gargling to prevent febrile diseases and sickness absences among healthy children. In their study, 19,595 children aged 2–6 years were observed for 20 d, and they found that fever onset was significantly lower in those that gargled with green tea (adjusted OR 0.32 95% CI 0.17–0.61) compared to those that gargled with tap water (adjusted OR 0.70; 95% CI 0.58–0.85) (reference, non-gargling group). Also, the prospective cohort study by Yamada et al. [40] studied the effect of gargling with green tea in 124 residents living in a nursing home for the elderly. They consumed the tea catechins three times daily for three months at a concentration equivalent to about half that of a commercially available green tea beverage with 200 μg per mL of total catechins. They found that the incidence of influenza decreased compared to gargling with water (incident rate; 1.3% in the green tea group vs. 10.0% in the water group). In their study, an influenza infection was determined based on a rapid antigen detection test, which is commonly used in clinical practice.

Three randomized controlled studies that evaluated gargling with green tea have been reported. Yamada et al. [41] reported on the association between gargling with green tea and the incidence of influenza infection. They randomly allocated 404 participants aged 20–65 years living in an elderly nursing home into the catechin group (gargling with extracts containing approximately 400 μg per mL catechins including 59.3% EGCG) or the placebo group. After three months, the incidence of influenza infection was 1.0% in the catechin group and 2.0% in the placebo group. Although the infection rate was lower in the catechin group than in the placebo group, the difference was not statistically significant (*p* = 0.84). Similarly, Toyoizumi et al. [33] and Ide et al. conducted a randomized controlled study on school-age populations [38,34]. They used bottled green tea solids that contained 560 μg total catechins per mL including 18% EGCG (N = 307 participants) or 370 μg total catechins per mL including 18% EGCG (N = 747), respectively. After three months, the incidence of influenza infection in either study was not significantly different between the green tea gargling group and the control group based on univariate and the multivariate analyses (*p* = 0.96 and *p* = 0.24, respectively) Recently, Ide et al. [42] reported on a meta-analysis that included randomized controlled studies and prospective cohort studies to assess the effect of gargling with tea on the prevention of influenza infection [32,33,34,35,36,37,38,39,40,41]. A total of 1890 subjects were pooled and analyzed, and the integrated participants who gargling with tea had a lower risk of influenza infection than participants who gargling with a placebo or who do not gargle (fixed effects model: relative risk (RR) = 0.70; 95% CI 0.54–0.89; random effects model: RR = 0.71, 95% CI 0.56–0.91). However, it should be noted that a limited number of studies were included in the analysis and the potential publication bias could not be evaluated.

Although there are reports that suggest the prevention of influenza and common cold by gargling, it should be noted that the mechanism of preventing these respiratory infections with gargling is unclear. Generally, it is thought that many causative bacteria and viruses of respiratory infections invade from the nasal cavity and infect the nasopharynx cells in several tens of minutes to hours. Therefore, it is hardly understood from the microbiological point of view as to how gargling, which is only carried out several times a day, acts prophylactically.

Although this has not been studied using green tea, regarding the acute upper respiratory tract infection effect of gargling with tap water, Satomura et al. reported that incidence rate had reduced to 36% in a cohort study involving adults [38]. Contrarily, a study conducted by Goodall et al. reported that gargling did not reduce the risk of upper respiratory tract infections in a randomized controlled study [43]. Therefore, a more detailed study is required to verify the effects of preventing influenza and common cold and whether this is the direct effect of gargle itself or the effect of catechin. In addition, to increase the clinical evidence or evaluate the beneficial effects, large-scale observational studies or randomized controlled studies are needed in the future.

## 6. Conclusions and Future Perspectives

Currently, research on the clinical effects of tea catechins on influenza and common cold is in the developing stage. As shown in Table 1, the results of two randomized control studies showed the clinical effects of tea catechins against influenza. Similar results were also reported in an observational study.

The results of three randomized control studies were not statistically significant; however, the result of a meta-analysis suggested that gargling with tea catechins confers lower risks of influenza infection.

In summary, although the number of clinical/epidemiological studies on tea catechins against influenza and common cold are limited, the present studies suggest the possibility of preventive effects on influenza and common cold.

A variety of experimental in vivo or in vitro studies on catechins and their chemical derivatives have been reported to have several anti-influenza virus effects, and three mechanisms have been proposed: (1) inhibition of attachment to the host cell, (2) replication inhibition, and (3) NA inhibition in the virus. In this review, the results of several clinical studies on green tea or its ingredients were briefly summarized. Although experimental studies indicated the antiviral activity of tea components, there is limited clinical evidence to support their utility for preventing influenza or the common cold. Because the reported clinical trials for the efficacy of catechins or other green tea ingredients were small-scale studies, it is difficult to deduce a clear conclusion. Additional large-scale observational studies or randomized controlled studies are needed to establish or confirm their clinical efficacy in humans in future studies.

In 2009, the world experienced a swine influenza virus pandemic. In addition, there are concerns about human infection with a highly pathogenic avian influenza virus that is prevalent locally. Furthermore, the prevalence of viruses that acquired resistance to existing NA inhibitors such as oseltamivir (Tamiflu) and zanamivir (Relenza) has also been confirmed. Under such circumstances, the development of new drugs that inhibit virus infection with different mechanisms of action are urgently needed. We hope that future studies and additional information will clarify the potential for catechins to serve as an effective antiviral therapy with the currently approved prescription drugs.

## Figures and Tables

**Table 1:** Characteristics of major studies on tea and its ingredients against influenza infection.

Source	Study Design/Observation Period	Analyzed Population	Observation Period	Measurement Outcomes
Park et al. 2011 [35]	Observational study	2050 primary school children; age, 6–13 years	-	Consumption of green tea prevented influenza infection. (1–3 vs. <1 cups per day, OR (95% CI) = 0.62 (0.41–0.95); 3–5 vs. <1 cups per day, OR (95% CI) = 0.54 (0.30–0.94))
Rowe et al. 2007 [36]	Randomized-controlled trial	108 healthy adults; age, 18–70 years	3 months	Fewer participants in the green tea capsule group showed symptoms compared with participants in the placebo group (63.6% vs. 43.2%, *p* = 0.035)
Matsumoto et al. 2011 [37]	Randomized-controlled trial	197 eligible healthcare workers; mean age, 42.7 years	5 months	Significantly lower incidence of influenza infection in the catechin group (4.1%) compared with the placebo group (13.1%) (OR = 0.25; 95% CI 0.07–0.76)

**Table 2:** Characteristics of major studies on gargling with tea and its ingredients for influenza infection.

Source	Study Design/Observation Period	Analyzed Population	Observation Period	Measurement Outcomes
Noda et al. 2011 [39]	Observational study	19595 children in nursery school; age, 2–6 years.	20 days	The low fever onset absence was associated with gargling with green tea in comparison with tap water. (OR (95% CI): 0.29 (0.16–0.55) in the green tea group, OR (95% CI): 0.74 (0.62–0.88) in the tap water group)
Yamada et al. 2006 [40]	Interventional study	124 nursing home residents; mean age, 83 years.	3 months	Significantly lower incidence of influenza infection in the group gargling with green tea than in the group gargling with water (OR = 15.7; 95% CI 1.88–399.7)
Yamada et al. 2007 [41]	Randomized-controlled trial	395 healthy adults; age, 20–65 years	3 months	Incidence of influenza infection was 1.0% of participants in the catechin group and 2.0% in the control group (P = 0.84)
Toyoizumi et al. 2013 [33]	Randomized-controlled trial	307 high school students; age, 15–17 years	3 months	Incidence of influenza infection was 7.1% of participants in the catechin group and 7.9% in the control group (P = 0.96)
Ide et al. 2014 [34]	Randomized-controlled trial	747 high school students; age, 15–17 years	3 months	Multivariate logistic regression indicated no significant difference; incidence of influenza infection was 4.9% in the green tea group and 6.9% in the water group (OR = 0.69; 95% CI 0.37–1.28)
